# Retraction: PATZ1 induces PP4R2 to form a negative feedback loop on IKK/NF-κB signaling in lung cancer
Retraction: MIG-7 and phosphorylated prohibitin coordinately regulate lung cancer invasion/metastasis

**DOI:** 10.18632/oncotarget.27233

**Published:** 2019-10-01

**Authors:** Ming-Yi Ho, Chi-Ming Liang, Shu-Mei Liang

**Affiliations:** ^1^ Genomics Research Center, Academia Sinica, Taipei 11529, Taiwan, ROC; ^2^ Agricultural Biotechnology Research Center, Academia Sinica, Taipei 11529, Taiwan, ROC

**These articles have been retracted:** On April 10, 2018, based on concerns regarding image duplication across different articles, Oncotarget requested that The Ethics Committee of the Division of Life Sciences of Academia Sinica initiate an investigation into two Oncotarget papers by Ho, et al (https://doi.org/10.18632/oncotarget.2804 and https://doi.org/10.18632/oncotarget.10427). After a series of Committee meetings, preliminary investigative reports, and multiple correspondence emails between the Committee and corresponding authors, the Committee made the following conclusions:

1. Four pairs of panels (2c, 9b), (3a, 8c), (3b, 8b) and (3d, 4c) in Fig. 7A, in https://doi.org/10.18632/oncotarget.10427, and two pairs of panels (1a,1c) (2c,3e) in Fig. 6A in https://doi.org/10.18632/oncotarget.2804 appear to be duplicated use of images from the same mice. The Committee finds that these results are falsified and may be misleading.

2. The corresponding authors of these papers, Drs. Chi-Ming Liang and Shu-Mei Liang, and the first author Ming-Yi Ho, all agreed that Dr. Ho is responsible for the execution of the experiments and providing the data. The committee concluded that Dr. Ho is thus responsible for research misconduct. Drs. Liang CM and Liang SM are responsible for the failure of proper mentoring/supervision.

